# Primary Pulmonary Synovial Sarcoma: A Very Rare Presentation

**DOI:** 10.1155/2014/537618

**Published:** 2014-08-03

**Authors:** Ekrem Cengiz Seyhan, Sinem Nedime Sokucu, Gulsah Gunluoglu, Nurdan Simsek Veske, Sedat Altin

**Affiliations:** ^1^Department of Chest Diseases, Medical Faculty, Medipol University, 34214 Istanbul, Turkey; ^2^Yedikule Teaching Hospital for Chest Diseases and Thoracic Surgery, Istanbul, Turkey

## Abstract

Synovial sarcoma (SS) is a rare tumor originating from mesenchymal tissue and accounting for approximately 5–10% of all soft tissue sarcomas. A rare case of primary pulmonary SS in an asymptomatic 18-year-old man admitted to our hospital for investigation of a 6 × 6.5 cm, oval-shaped, well-delineated pleural based peripheral mass in the left lower lobe in his thorax CT is presented. Left lower lobectomy was done. Immunohistochemically, tumor cells were positive for cytokeratin, epithelial membrane antigen (EMA), and vimentin so that the histopathological diagnosis was compatible with biphasic spindle cell type SS in the lung.

## 1. Introduction

Synovial sarcomas (SS) account for nearly 5–10% of all soft tissue sarcomas [1, 2]. Clinically it is presented as a palpable and painful soft tissue mass. SS not only occurs predominantly in the large joints of the extremities, but also could occur in neck, tongue, larynx, mediastinum, esophagus, heart, lung, abdomen wall, small intestine, mesentery, vessels, and retroperitoneum. Thoracic involvement of the SS in the literature was reported to be very rare [1, 2]. Primary pulmonary and mediastinal SS is an aggressive tumor sharing common histological features with soft tissue SS [3, 4]. SS, although rare, is a primary pulmonary and mediastinal neoplasm with distinctive histology. It's rare occurrence in this region cause to be overlooked in the differential diagnosis. Recognizing radiological, histopathological, and molecular properties of the SS is very important for appropriate treatment.

## 2. Case Report

An 18-year-old man was referred for investigation of a peripheral opacity in the left lung lower lobe, which was discovered incidentally on a chest radiograph. The patient is a student and has a 6-pack/year smoking history. He was in good general health and well nourished. Physical examination was normal. The results of blood tests and standard biochemical tests were normal.

**Figure 1:**
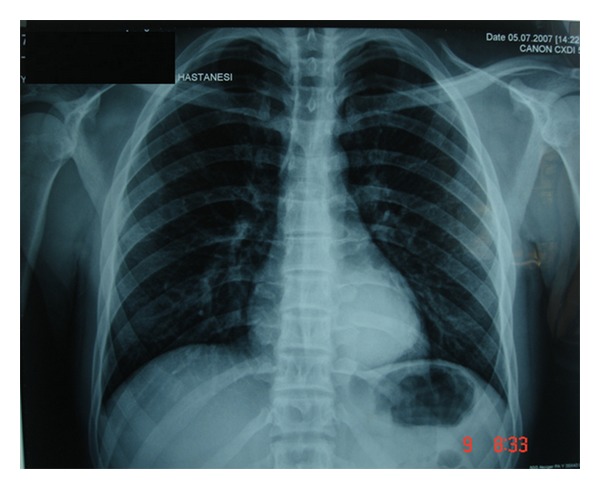
PA chest X-ray revealed a well-demarcated 6 cm peripheral opacity in the left lower lobe nearly by the diaphragm.

Posteroanterior chest X-ray (Figure 1) revealed a well-demarcated 6 cm in diameter peripheral opacity in the left lower lobe near by the diaphragm. Chest computed tomography (CT) confirmed a 6 × 6.5 cm, oval-shaped, well-delineated pleural basedperipheral mass in the left lower lobe, in soft tissue attenuation and with no evidence of mediastinal or axillary adenopathy. Cyst hydatid hemagglutination was negative. Fiberoptic bronchoscopy showed no endobronchial pathology. Bronchoalveolar lavage and bronchial brushing specimens, obtained during bronchoscopy, were negative for malignancy.

**Figure 2:**
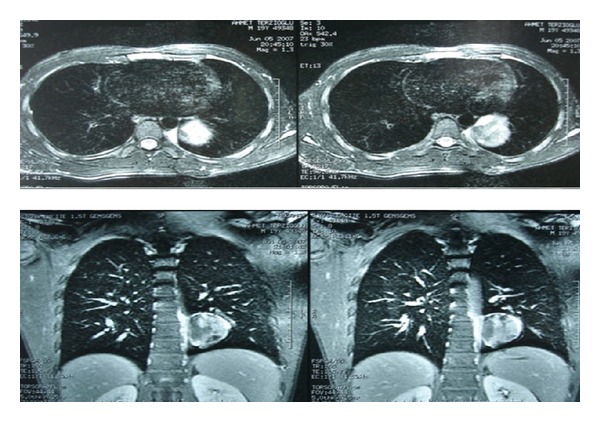
On the thorax MR angiography a 6 × 6.5 cm, oval-shaped, pleural based mass in the left lower lobe was seen.

Thorax CT angiography was taken to reveal vascular relation of the tumor. Heterogeneous contrast enhanced 6,5 × 4,5 × 6 cm mass lesion located at posterobasal segment of the left lung lower lobe was seen (Figure 2). No vascular relation was detected. The CT-guided fine needle aspirate from the mass revealed roundcell tumor. Full body bone scintigraphy and cranial magnetic resonance imaging (MR) taken for metastasis evaluation were normal.

**Figure 3:**
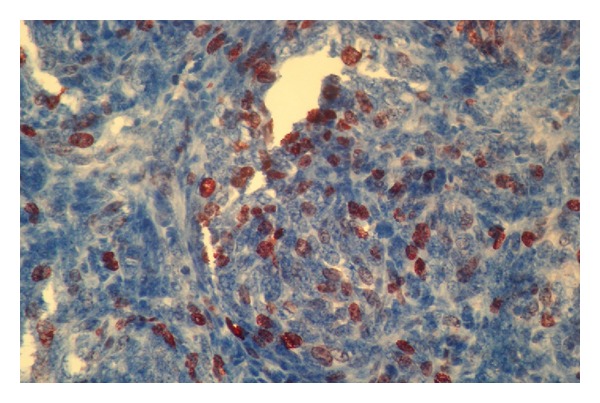
Immunohistochemically tumor cells were positive for cytokeratin and vimentin.

Left posterolateral thoracotomy was performed. At lower lobe of the lung a big tumor at posterobasal segment was observed. The tumor was under the visceral pleura. Subsequently left lower lobectomy was done with dissection of the mediastinal lymph nodes. Histopathological evaluation revealed well-circumscribed nodular 8 × 6.5 × 6 cm mass. The tumor was adjacent to the visceral pleura but did not invade it. Microscopically the tumor was having spindle cells arranged in a dense cellular network. Immunohistochemically, tumor cells were positive for cytokeratin, epithelial membrane antigen (EMA), and vimentin so that the histopathological diagnosis was compatible with biphasic spindle cell type SS in the lung (Figure 3). A molecular analysis performed using reverse transcriptase-polymerase chain reaction with RNA (RT-PCR) extracted from paraffin-embedded tissue to confirm the diagnosis revealed SYT-SSX1 fusion gene.

After positron emission tomography/computed tomography with 18F-fluorodeoxyglucose (^18^F-FDG PET/BT) evaluation and oncological and orthopedic consultations, SS was accepted to be primary pulmonary. He received 3 courses of chemotherapy (adriblastina and ifosfamide). One year later two nodules, one at residual left upper lobe and a second at right lower lobe, occurred. The nodules were resected sequentially via wedge resections of the lobes. At the third year of followup, second recurrence occurred at residual left upper lobe. Due to small volume of the residual left lung, completion left pneumonectomy with chest wall resection was done. After the completion pneumonectomy the patient received adjuvant chemotherapy for six courses with adriblastina and ifosfamide. One year later locoregional recurrence at the pneumonectomy area and also a nodular metastasis at the right lower lobe superior segment and vertebral metastasis at T6–T8 vertebrae were detected. Then, the patient received radiotherapy (10 × 300 cGy) for vertebral metastasis. The patient died after two months.

## 3. Discussion

SS is a rare tumor originating from mesenchymal tissue and accounting for less than 0.5% of all soft tissue sarcomas [1, 2]. Age distribution in patients with SS is higher in comparison with other sarcomas. It was usually seen in between 3rd and 5th decades and has an almost equal gender distribution [4, 5]. Most common presentation is with chest pain [4]. In the presented case lesion was detected as an incidental finding in chest X-ray.

The presented case has a well-delineated peripheral mass in the chest X-ray which was compatible with the earlier reported series done by Frazier et al. in which 78% of the cases have lesions with well-delineated borders [6]. Also the lesion has no calcification or cavitations which were consistent with the same series of results and the other hemithorax was normal. CT demonstrates a well-defined homogeneous or heterogeneous enhancing mass containing necrotic areas and soft tissue components [4]. In the CT evaluation of the case, a wide pleural based localization of the mass was reported to be seen as in 73% of the cases by Frazier et al. [6]. Ipsilateral pleural effusion is common, which was not seen in our case. In our case, no bone destruction was detected but the tumor located intrathoracically and extrapulmonary has extension to the chest wall. No chest wall invasion was documented in the series by Frazier et al. while Tateishi et al. reported that infiltration to the adjacent tissue could occur [6, 7]. Radiologically, compared with soft tissue SS, primary pulmonary and mediastinal SS shows less vascularity, hemorrhage, and necrosis on MR [4]. As in our case no vascular relation was detected in CT angiography of the lesion.

Tumor does not originate from the synovial membranes but just resembles synovial tissue tumor under the light microscopy [1, 2]. Histologically tumor could be monophasic or biphasic type composed of epithelial and spindle cells with different ratios. Immunohistochemically most synovial sarcomas were positive for vimentin, cytokeratins, and EMA and also could show lower immunoreactivity for S-100 and CD34 [3]. As in our case vimentin, cytokeratins, and EMA were positive. However, immunohistochemical staining cannot distinguish SS from the mesothelioma. So, a confirmation test is needed. In more than 90% of cases a specific chromosomal fixed translocation t(X; 18) (p11.2; q11.2) was detected. RT-PCR was performed for detection of t(X; 18) (p11; q11) translocation fusion product [8, 9]. This gene expression pattern is important in verifying the diagnosis and also in predicting the prognosis [9]. SS with the SYT-SSX1 fusion transcript (irrespective of the histological type) has a poorer prognosis than cases with SYT-SSX2 fusion transcript and respective 5-year progression-free survival rates of 42% versus 89% [9]. In the presented case SYT-SSX1 fusion transcript was also detected which could be confirming the diagnosis. Additionally, in our case at preoperative bone scintigraphy and postoperative whole body PET-CT, no other lesion was detected; also histopathological evaluation showed no visceral pleura involvement, so the tumor was accepted as primary pulmonary SS.

Treatment of these tumors is wide surgical resection with removal of the tumor with free surgical margins with or without radiotherapy for local control. Adjuvant chemotherapy for soft tissue sarcoma is controversial [3, 10]. Cure was related to how radical the resection is. Recurrences could be seen [7]. Wide surgical resection and chemotherapy were done and patient also underwent recurrent surgery due to recurrences occurring every other year for 3 years.

In conclusion primary pulmonary SS is a rare and aggressive tumor. It should be diagnosed with radiological properties, histological methods, and molecular techniques as early as possible after other alternative diagnoses were excluded and wide resections were needed for the treatment of these tumors. Also due to high risk of recurrences, a long-term followup will be needed.
